# Evaluation of Risk Factors Associated with Expectant Management in CIN 1/2: A Multicenter Real-World Cohort Study

**DOI:** 10.3390/cancers17233738

**Published:** 2025-11-22

**Authors:** Sanha Lee, Heekyoung Song, Hong Yeon Lee, Sujin Lee, Jeongyoon Lee, Suein Choi, Soo Young Hur

**Affiliations:** 1 Department of Obstetrics and Gynecology, Seoul St. Mary’s Hospital, College of Medicine, The Catholic University of Korea, Seoul 06591, Republic of Korea; sanhalee0327@catholic.ac.kr (S.L.); snujin94@gmail.com (S.L.); 2 Department of Obstetrics and Gynecology, Incheon St. Mary’s Hospital, College of Medicine, The Catholic University of Korea, Seoul 06591, Republic of Korea; songdeng86@catholic.ac.kr; 3 Department of Obstetrics and Gynecology, Yeouido St. Mary’s Hospital, College of Medicine, The Catholic University of Korea, Seoul 06591, Republic of Korea; lhy09091414@gmail.com; 4 Department of Pharmacology, College of Medicine, The Catholic University of Korea, Seoul 06591, Republic of Korea; hansnuna@gmail.com; 5 Division of Data Science, Pharmacometrics Institute for Practical Education & Training, College of Medicine, The Catholic University of Korea, Seoul 06591, Republic of Korea

**Keywords:** cervical intraepithelial neoplasia, CIN 1, CIN 2, human papillomavirus, expectant management, risk factors

## Abstract

**Background/Objectives:** To evaluate the effectiveness of expectant management on grades 1 and 2 cervical intraepithelial neoplasia (CIN), including factors associated with regression and progression. **Methods:** This multicenter study included 561 women managed expectantly and 359 who underwent immediate surgery at eight institutes between 2013 and 2023. **Results:** Over a 4-year period, 63% and 68% of CIN 1 and CIN 2 cases regressed, and 9% and 14% of cases progressed, respectively. The median regression times were 1.5 years for CIN 1 and 1.2 years for CIN 2. High-risk human papillomavirus (HPV) types, especially HPV 58 (adjusted hazard ratio [HR]: 0.61; *p* = 0.032) and high-grade initial cytology, atypical squamous cells—cannot exclude high-grade squamous intraepithelial lesion (ASC-H) and high-grade squamous intraepithelial lesion (HSIL) (adjusted HR: 0.3, *p* < 0.001), were associated with a lower likelihood of regression. Also, hematological disorders reduced the likelihood of regression (adjusted HR 0.39, *p* = 0.045). In a separate analysis of the immediate surgery group, age in the 30s (*p* = 0.016) and HPV 16 infection (*p* = 0.005) were associated with pathologic upgrading at surgery. **Conclusions:** CIN 1 and 2 usually regress, allowing expectant management for up to 1.5 and 1.2 years, respectively. However, HPV 58 infection or high-grade initial cytology, and hematological disorders are indications for careful monitoring. Patients in their 30s or infected with HPV 16 have a higher risk of pathologic upgrading at surgery.

## Simple Summary

Cervical intraepithelial neoplasia (CIN) is a precancerous cervical lesion that may regress or progress to cancer. Clinicians must decide between surgical treatment and expectant management, but predicting safe observation remains difficult. Using multicenter data, we examined how age, human papillomavirus (HPV) infection, and blood disorders influence CIN outcomes. Most CIN 1 and CIN 2 lesions regressed spontaneously, while high-risk HPV types, abnormal cytology, and hematological disorders decreased the likelihood of regression. These findings may inform clinical decision-making and follow-up strategies for precancerous lesions of the cervix.

## 1. Introduction

In 2022, cervical cancer was associated with an estimated 660,000 new cases and 350,000 deaths worldwide [1]. In South Korea, the age-standardized incidence rate of cervical cancer per 100,000 people was 3.7 in 2020 [2]. Cervical cancer is associated with a persistent human papillomavirus (HPV) infection, which can lead to the development of precancerous lesions that may progress to invasive malignancy [3]. Cervical cancer screening programs that include regular follow-up HPV tests or cytology and HPV prophylactic vaccination programs have been implemented globally. The incidence of cervical cancer has decreased in Sweden [4] and South Korea from 8.6 per 100,000 in 1999 to 4.4 per 100,000 in 2018 [5].

However, precancerous lesion incidence increased from 17,651 in 2018 to 19,464 in 2022 [6], suggesting the need to manage these lesions to help prevent new cancer cases. The current guidelines recommend more frequent screening, including cervical tissue biopsies, and treatment for patients at a higher risk such as those with high-grade squamous intraepithelial lesions (HSIL). Patients with lower risk profiles, such as those with low-grade squamous intraepithelial lesions (LSIL), can undergo follow-up tests at longer intervals.

Cervical excision is associated with increased risks of preterm birth and cervical stenosis [7]; however, delayed loop electrosurgical excision procedure for cervical intraepithelial neoplasia (CIN) 2 may increase the risk of preterm birth by 30% [8]. Thus, strategies to identify patients most likely to benefit from immediate treatment and those who may require expectant management are needed. Even though excision treatment should be applied in the precisely needed case, Korean clinicians prefer to perform cervical excision in CIN patients owing to the risk of cancer. Therefore, this study used real-world data to identify patients with CIN and the disease characteristics associated with regression, helping to inform future follow-up and treatment strategies. In addition, this study aimed to characterize HPV genotype–specific regression patterns of CIN in Korean women, providing preliminary evidence that may support future prophylactic vaccination strategies.

## 2. Materials and Methods

This study was designed a retrospective, observational, multicentric cohort study. Medical records of patients who underwent cervical cancer screening were obtained from the clinical data warehouse, which encompasses 8 hospitals in Catholic University of Korea and contains approximately 15 million fully anonymized electronic medical records, enabling data extraction based on search criteria. The study protocol was approved by the Institutional Review Board (KC23WIDI0321). The requirement for informed consent was waived owing to the retrospective study design and use of fully anonymized data.

### 2.1. Study Cohort

We included participants with biopsy-confirmed precancerous cervical lesions (between 1 January 2013 and 14 February 2023). The primary cohorts were defined as the CIN 1 and CIN 2 groups, based on pathological results obtained at the first visit. Only patients with formal pathology reports issued within the Catholic Medical Center network—comprising secondary and tertiary care institutions that have pathology departments accredited by the Korean Society of Pathologists quality control program and operate collaboratively as teams—were eligible for inclusion. In this network, both internal and external consultations are routinely conducted, ensuring high diagnostic reliability. To ensure reliable outcome assessment and minimize misclassification of transient cytologic or virologic changes, patients were required to have a minimum follow-up duration of 365 days and at least two follow-up visits approximately six months apart to confirm sustained clinical outcomes. The exclusion criteria were as follows: confirmed or diagnosed cervical cancer at the first visit; history of hysterectomy for another gynecologic disease; missing pathological results; any other diagnosed malignancy; and follow-up shorter than 365 days without at least two follow-up visits approximately six months apart.

Among the above patients, those who underwent expectant management were designated as the follow-up cohort. Cytology and HPV tests were typically performed at intervals of 6 months to 1 year. A repeat biopsy was conducted if the clinician identified potential signs of progression, such as changes in cytology, persistent high-risk HPV infection, or abnormal colposcopic findings. Surgical treatment was initiated if disease progression was confirmed or suspected due to persistent lesions.

To evaluate the effectiveness of immediate surgical treatment in the CIN 1 or CIN 2 groups, an additional cohort was created including patients who underwent surgical treatment within 1 year of diagnosis and excluded from the primary cohort. The disease codes used in the search for this study are detailed in Table S1. Sexually transmitted infections were confirmed based on the recorded prescriptions—missing information was confirmed through a chart review performed by a gynecologic oncologist.

### 2.2. Variables

CIN was categorized as cervical dysplasia based on nuclear abnormalities, abnormal patterns of cell division, loss of epithelial differentiation, and cellular tissue collapse [9]. According to the current terminology guidelines [10], HPV infection and CIN 1 may be difficult to distinguish histologically; therefore, they are classified as LSIL. In contrast, CIN 2 and 3 are classified as HSIL. The use of p16 immunohistochemical staining helps differentiate between precancerous lesions and infections [10]. This study retrospectively reviewed medical records beginning in 2010. For the initial cohort, only patients diagnosed with LSIL (CIN 1), HSIL (CIN 2), or HSIL (CIN 3) through biopsy were included, and only those with clear differentiation between CIN 2 and CIN 3 were eligible for inclusion.

The classification of LSIL (CIN 1), HSIL (CIN 2), and HSIL (CIN 3) was based exclusively on final pathology reports issued by board-certified pathologists at each participating institution. For CIN 1, particular care was taken to differentiate these lesions from chronic cervicitis or other benign inflammatory conditions, which were excluded from the cohort. These inflammatory conditions are typically characterized by negative p16 staining and lack of morphologic atypia. p16 immunohistochemistry was actively utilized to aid in the distinction between CIN 1 and CIN 2, particularly in cases with borderline histologic features. Among 1073 biopsy specimens from patients classified as CIN 1 and 775 from those classified as CIN 2, p16 staining was performed in 83.7% and 87.2% of cases, respectively, to improve diagnostic accuracy.

### 2.3. Progression and Regression

The primary outcomes of interest in this study were CIN progression and regression, as defined by changes in histological findings over time. Progression was defined as an increase in lesion severity, such as from CIN 2 to CIN 3, while regression was defined as a return to normal histology. Participants whose disease status remained unchanged were categorized as having persistent disease. We additionally defined partial regression as cases in which (1) HPV infection persisted despite normalization of cytology, or (2) histologic findings showed improvement (e.g., from CIN 2 to CIN 1), without complete clearance of HPV and normalization of cytology. This study did not account for any changes detected within the first month of diagnosis. In the absence of biopsy results, only two consecutive negative cytology and HPV test results were considered evidence of disease regression; otherwise, the case was classified as persistent (persistent or partial regression).

### 2.4. Definitions of Evaluated Risk Factors

Data on baseline characteristics were collected at the time of diagnosis. Initial HPV test and cytology results were recorded at the time of the biopsy, as well as within a three-month window before and after the procedure. If results from external institutions were available, they were verified through chart review and included accordingly. Cytology tests included both liquid-based cytology performed in routine outpatient clinics and conventional smear-based cytology conducted at health screening centers. HPV tests conducted at the eight institutes (Cobas HPV Amplification by Roche Diagnostics, Basel, Switzerland, Allplex™ HPV28 Detection by Seegene Inc., Seoul, Republic of Korea, etc.) received approval from the Korean Ministry of Food and Drug Safety. The number of HPV were categorized into high-risk types (16, 18, 26, 31, 33, 35, 39, 45, 51, 52, 53, 56, 58, 59, 66, 68, 69, 70, 73, 82) [11,12], low-risk types (6, 11, 30, 32, 34, 40, 42, 43, 44, 54, 55, 61, 62, 67, 74, 81, 83, 84, 87, 90), and “negative”, depending on the HPV profile. However, only the most clinically relevant 9-valent HPV (16, 18, 31, 33, 45, 52, 58) [13], and other high-risk HPV variants (35, 39, 51, 56, 59, 66, 68) were used as covariates in this analysis [12].

### 2.5. Statistical Analysis

Patient baseline characteristics were compared among the disease categories. Non-normally distributed variables were compared using the Kruskal–Wallis test. Categorical variables were compared using Fisher’s exact test. Continuous and categorical variables are expressed as medians with ranges and frequencies or percentages, respectively.

The cumulative incidence functions for progression and regression were estimated, treating them as competing risks. Time-to-event was calculated from baseline to progression, regression, or death, with participants censored at their last visit if they had persistent disease or had died. Gray’s test was employed to assess differences in cumulative incidence rates, and the Gray sub-distribution hazard model to estimate hazard ratios (HRs) and 95% confidence intervals (CIs) for risk factors. Separate multivariate models were built for each high-risk HPV variant due to limited events. Multicollinearity was checked using the variance inflation factor, excluding covariates with values ≥ 5. All analyses were conducted using R (version 4.3.3; R Foundation for Statistical Computing, Vienna, Austria) with the “tidycmprsk” package, considering *p*-values < 0.05 as statistically significant.

## 3. Results

We identified 617 cases of CIN 1, including 469 cases that were followed up for at least 1 year, resulting in 294 (63%), 131 (29%), and 20 (9%) cases of complete regression, persistent disease, and progression, respectively. In addition, we identified 339 cases of CIN 2, including 92 that were followed up for more than 1 year, resulting in 63 (68%), 16 (17%), and 13 (14%) cases of complete regression, persistent disease, and progression, respectively. This cohort was designated as the expectant management group (referred to as the follow-up cohort in Figure S1).

The participants’ characteristics are presented in Table 1. At least one high-risk HPV type was detected in 324 (61%) patients, and it was more common in cases of progression (84%) than in cases of persistent disease (59%) or regression (58%) (*p* < 0.001). HPV types 16 and 58 were most common, especially in the progression cases (*p* < 0.05). A majority of HPV-negative patients (91 out of 118, 77%) showed lesion regression. Meanwhile, HPV type 31 was rare in the regression group (*p* < 0.05). Several high-risk HPV types were co-detected in 14% of the cases. High-risk initial cytology [atypical squamous cells—cannot exclude high-grade squamous intraepithelial lesion (ASC-H) or HSIL] accounted for the smallest proportion of cases in the regression group (4.5%) but the largest proportion in the progression group (25%). However, cases with low-risk initial cytology still made up 75% of those that progressed. Hematological disorders, such as anemia or thrombocytopenia, or tumors of the hematopoietic and lymphoid tissues, were observed in 14 (2.5%) of all patients and were more prevalent in the progression group (7.5%, *p* = 0.022) than in the other groups. Pelvic inflammatory disease was present in 260 (46.4%) patients, but it did not affect outcomes (*p* = 0.7).

**Figure 1:**
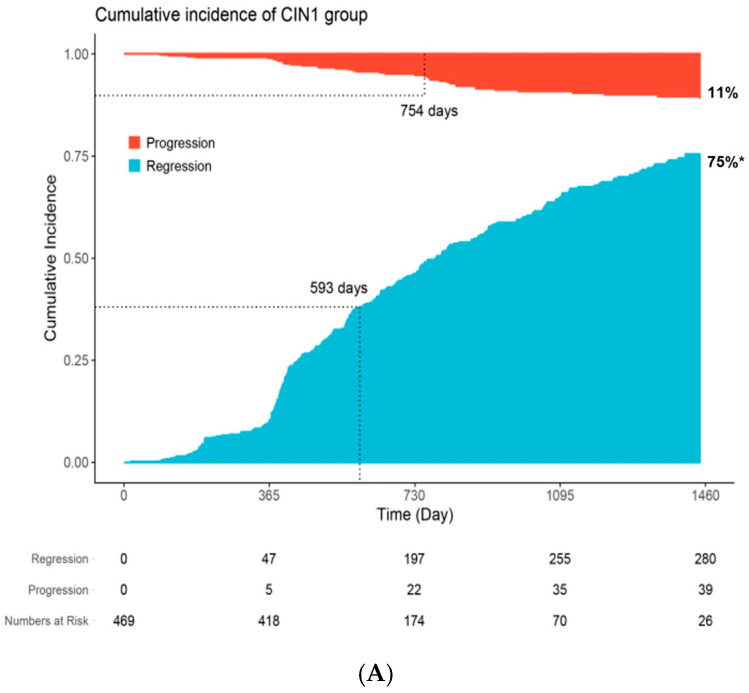

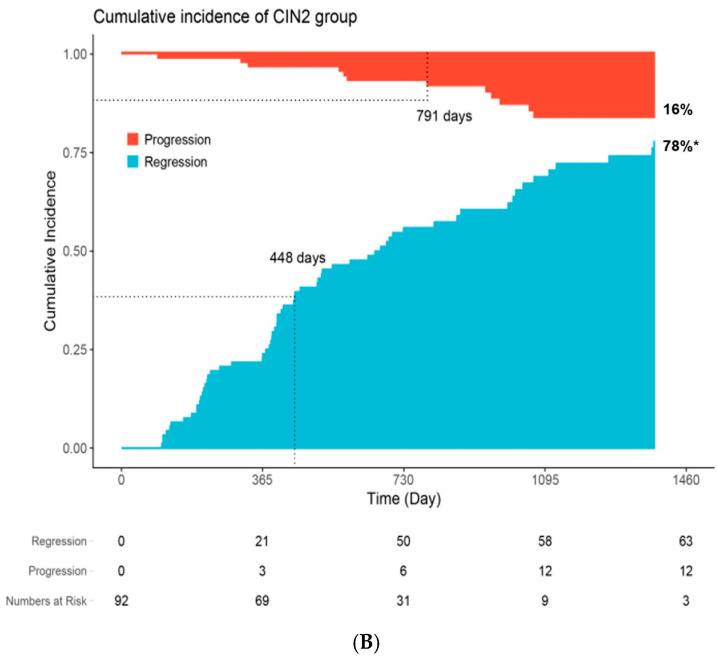
Cumulative incidence of lesion regression and progression over 4 years among patients with CIN1 (**A**) and CIN2 (**B**). * Curves represent time-to-regression estimated using the cumulative incidence function, accounting for competing risks, including death and loss to follow-up. Regression was defined as histologic or cytologic resolution without progression. The cumulative probability (75% and 78% at 4 years, respectively) differ from the crude regression proportions (63% and 68%, respectively) reported in the abstract, which do not account for time-to-event or censoring.

Regarding cumulative incidence of regression and progression, in the CIN 1 group (Figure 1A), 75% and 11% of cases showed regression and progression, respectively, during the 4-year follow-up. The median times to regression and progression were 593 and 754 days, respectively, indicating that 50% of the events occurred before this time point during the follow-up period. In the CIN 2 group (Figure 1B), regression and progression were observed in 78% and 16% of the cases, respectively, during the same period. The median times to regression and progression were 448 and 791 days, respectively.

Table 2 and Table 3 summarizes the univariate and multivariate analyses of factors influencing CIN regression using a sub-distribution hazard model. Patients with high-grade initial cytology (ASC-H/HSIL) were less likely to experience regression with a significantly lower regression rate (adjusted HR: 0.3, *p* < 0.001). Also, hematological disorders decreased regression likelihood (crude HR: 0.39, *p* = 0.035; adjusted HR: 0.39, *p* = 0.045). Nutritional anemia (D50–53) accounted for 77% of patients with hematological disorders, and hemolytic, aplastic, or other diseases (D55–89) accounted for the remaining 23% (Table 2). Between the bad prognosis group (persistent or progression in the observation group and worse-than-expected outcomes in the early intervention group) and the good prognosis group (regression in the observation group and expected outcomes in the early intervention group), the absolute neutrophil count (ANC) was lower in the bad prognosis group (mean ANC = 3001) than in the good prognosis group (mean ANC = 5772; *p* = 0.043). In contrast, lymphocyte [%] tended to be higher in patients with a good prognosis than in those with a poor prognosis (median 41% vs. 28%), although this difference did not reach statistical significance (*p* = 0.14) (Table S2). High-risk HPV infection was associated with a lower likelihood of regression (crude HR: 0.78, *p* = 0.020; adjusted HR: 0.78, *p* = 0.025). Although multiple HPV coinfections showed a similar trend toward reduced regression, the association did not reach statistical significance (crude HR: 0.65, *p* = 0.011; adjusted HR: 0.73, *p* = 0.066). Among individual genotypes, HPV 58 was independently associated with a lower likelihood of regression (adjusted HR: 0.61, *p* = 0.032) (Table 3). The same risk factors were identified in multivariate analyses for progression as those for regression outcomes. Compared that, HPV types 16, 33, and 58 were identified as significant risk factors for progression (adjusted HR: 3.15, *p* = 0.004; adjusted HR: 5.25, *p* = 0.028; adjusted HR: 3.14, *p* = 0.002, respectively) (Table S3).

**Figure 2:**
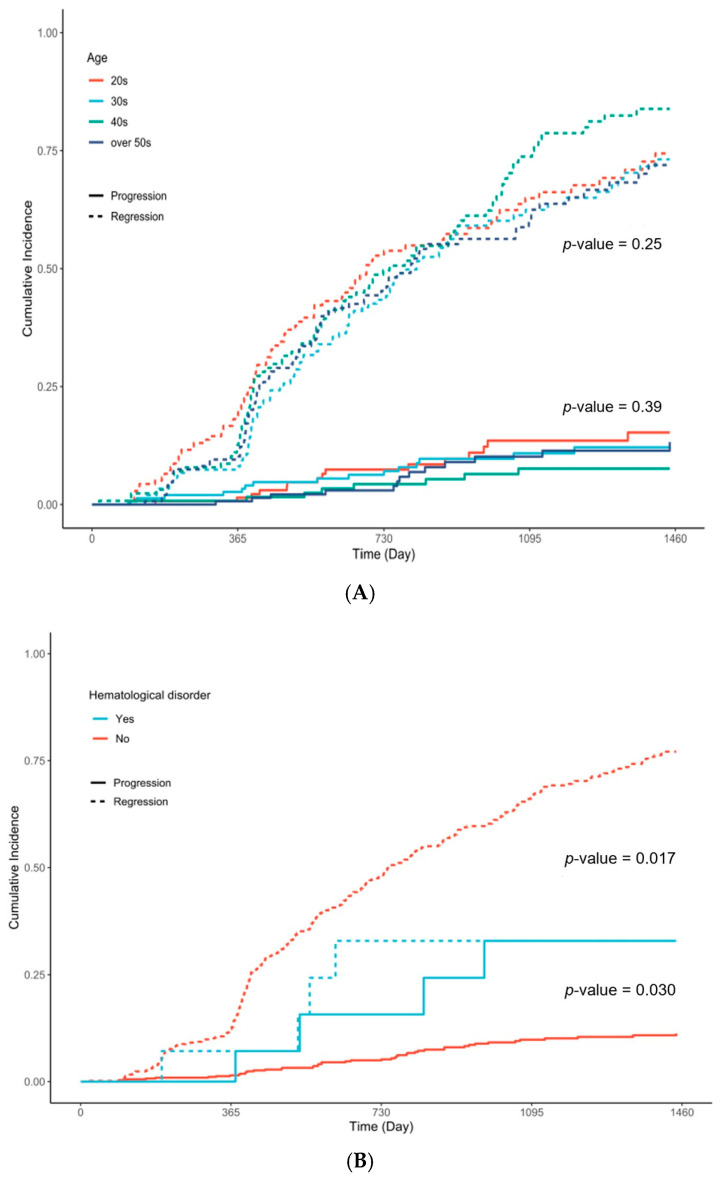

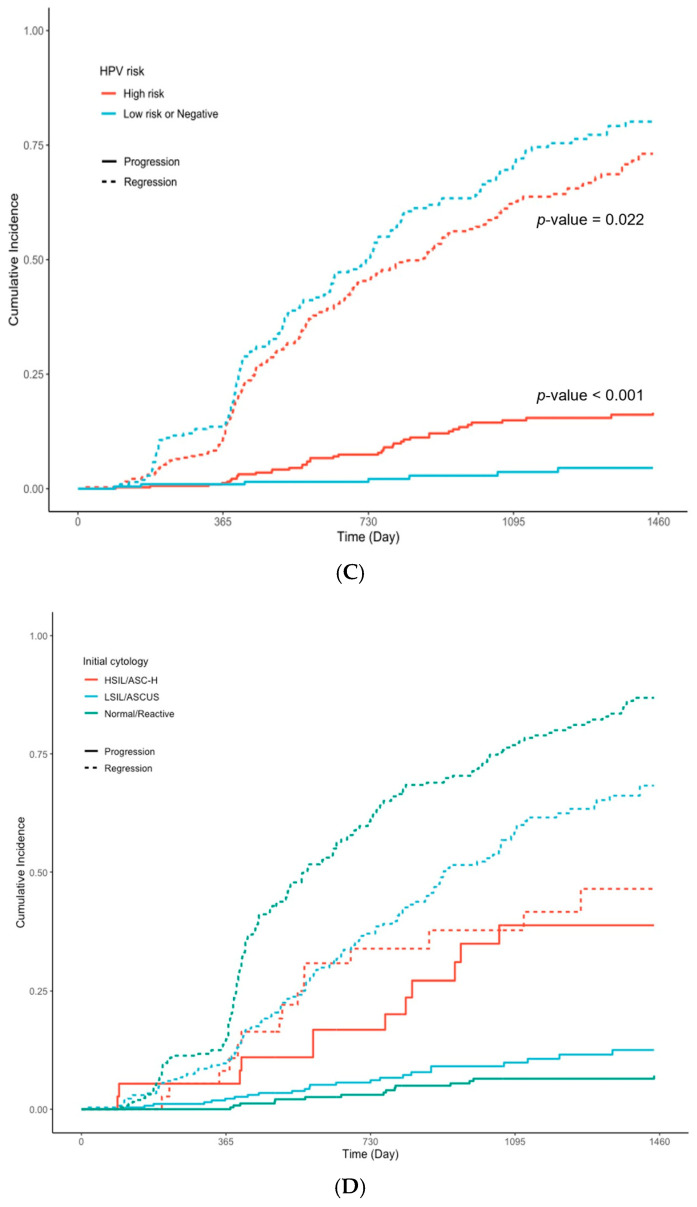
Cumulative incidence rates of progression and regression by risk factors: (**A**) age (**B**) hematological disorder (**C**) human papilloma virus (HPV) infection (**D**) initial cytology.

Figure 2A shows the incidence rates of progression and regression by age group, but the differences in clinical outcomes by age were not statistically significant. In contrast, hematological disorders (Figure 2B) significantly affected regression rates (*p* = 0.030). Additionally, high-risk HPV types and high-risk initial cytology were associated with reduced regression rates (*p* = 0.022 and *p* < 0.001, respectively) (Figure 2C,D).

The actual and expected outcomes of patients undergoing CIN surgical treatment based on pre- and post-operative biopsy results are presented in Table 4. A significant deviation from expected outcomes was observed in the “upgraded” pathology category, where almost 34% of the patients had a more severe diagnosis that was not indicated in the initial biopsy, and hidden CIN 3 was found in 26% of early intervention cases; patients with the initial diagnosis of CIN 2 were more likely to progress to CIN 3 than those with the diagnosis of CIN 1 (*p* < 0.001). Age (30s) was a significant risk factor for hidden CIN 3 (*p* = 0.016). In addition, HPV type 16 was associated with risk of hidden CIN 3 (*p* = 0.005).

## 4. Discussion

### 4.1. Summary of Main Results

In this study, we found that CIN 1 and CIN 2 typically regress, supporting expectant management for up to 1.5 and 1.2 years, respectively. However, high-risk HPV infection—particularly HPV 58—high-risk initial cytology, and hematological disorders were associated with a lower likelihood of regression and warrant closer monitoring. Additionally, patients with a bad prognosis had lower absolute neutrophil counts (*p* = 0.043).

In a separate analysis of the immediate surgery group, age in the 30s (*p* = 0.016) and HPV 16 infection (*p* = 0.005) were associated with pathologic upgrading at surgery.

### 4.2. Results in the Context of Published Literature

#### 4.2.1. Findings from the Expectant Management Group

We found that the regression rate for the CIN 2 group was 68%, supporting expectant disease management over a 4-year-period. A retrospective study conducted in New Zealand showed a regression rate of 64% [14] in CIN 2 cases, with a meta-analysis reporting a pooled regression rate of 50–55% [15,16]. The regression rate of this study was higher than that of other studies, which may be associated with selection bias whereby CIN 2 cases recommended for monitoring, rather than immediate surgery, are likely to represent small lesions. Approximately 73% of participants with CIN 2 underwent immediate surgery, with the cases referred for expectant management likely presenting with narrow colposcopy lesions, previously linked to regression [17].

Among CIN 1 cases, the regression rate was 63%, which is relatively lower than that reported in other studies (60–80%) [16,18]. The patients in this study were primarily from secondary or tertiary medical institutions in Korea rather than primary care facilities. Consequently, the outcomes of many CIN 1 patients who experienced regression may not have been fully captured. However, a meta-analysis reported an average regression rate of 60% [16], and a previous Korean HPV cohort study, which included participants comparable to those in the present CIN 1 group, reported a cytologic regression rate of 50% [19]. Therefore, the observed regression rate of CIN 1 falls within an acceptable range and suggests potential effects of population-level characteristics.

In the CIN 1 group, the median time to regression was 593 days (1.5 years), while the median time to progression was 754 days (2 years). In the CIN 2 group, the corresponding values were 448 days (1.2 years) and 791 days (2.2 years), respectively, suggesting that regression generally occurs earlier than progression. A large-scale study conducted in Denmark reported evidence of progression among CIN 2 patients at one-year follow-up [14], a shorter interval than observed in our study, highlighting the potential impact of population-level differences on CIN 2 progression risk. For CIN 1 cases, our findings support an observation period of up to 1.5 years before considering active treatment, which aligns with the current Korean guidelines for early detection of cervical cancer [20].

HPV genotype and initial cytology were strong predictors of clinical outcomes. Specifically, HPV 58 was associated with a lower likelihood of regression, while HPV 16 and 33 were linked to disease progression—highlighting the importance of genotype-based risk stratification, particularly in East Asian populations [21,22,23]. High-grade cytology (ASC-H/HSIL) was also significantly associated with reduced regression, reinforcing the value of combining cytologic and genotypic information in clinical decision-making.

The relatively high proportions of HPV-negative (22%) and cytology-negative (46%) cases in our cohort likely reflect the retrospective study design, where test results obtained within three months before or after biopsy were used as baseline values. This approach may have captured transient infections or cases that regressed spontaneously. Although the overall high-risk HPV detection rate in our study was 61%—lower than the 91.5% reported in a nationwide Korean study [24]—it aligns with international data, such as a Chinese study reporting 66.7% prevalence in CIN 1 cases [25]. Cytology-pathology discordance, especially in CIN 1 cases with high-grade cytology, has also been described by Ouh YT et al. [26], supporting American Society for Colposcopy and Cervical Pathology (ASCCP) guidelines recommending up to three years of follow-up even after negative results in such cases [27].

Hematological disorders such as anemia, Fanconi anemia, or thrombocytopenia emerged as factors that reduced regression rates in patients with HPV infection. Nevertheless, these disorders are relatively rare, and the evidence regarding their effects on HPV infection progression remains limited. In this study, ANC was identified as a potential prognostic factor for regression, and the effects of these comorbidities on the immune system may inhibit spontaneous HPV clearance. Experimental studies have also shown that genetic defects in Fanconi anemia can enhance the activity of HPV oncogenes, supporting the possible link between hematological immune dysfunction and viral persistence [28].

In addition, lymphocyte counts in our cohort showed a non-significant trend toward higher levels in patients with favorable outcomes, which may be attributable to the small sample size. As lymphocytes play a key role in HPV clearance, decreased levels may weaken immune surveillance and contribute to viral persistence [29]. Taken together, these findings suggest that patients with hematological disorders or lymphopenia may benefit from more frequent HPV follow-up testing and screening, although further large-scale studies are warranted to validate these observations.

To ensure the robustness of our findings, we performed two additional sensitivity analyses using logistic regression models. First, as suggested by the reviewer, we re-analyzed the data including only biopsy-confirmed regression cases (Table S3). In this “biopsy-only” analysis, the number of regression events markedly decreased from 357 to 174, resulting in reduced statistical power. Nevertheless, CIN 2 cases continued to show a higher likelihood of regression than CIN 1 cases, and patients with hematological disorders showed a non-significant but consistent trend toward lower regression rates.

Second, to address the concern that early regression events might have been missed, we conducted another logistic regression analysis restricted to participants with ≥6 months of follow-up, which slightly increased the total number of eligible patients from 561 to 588. In this analysis, the trend of higher regression in CIN 2 cases compared with CIN 1 was maintained but did not reach statistical significance. Likewise, the associations for HPV 58 infection and hematological disorders were non-significant, although their directions were consistent with the main analysis (Table S4).

Taken together, these complementary logistic analyses indicate that overly stringent definitions or inclusion criteria may reduce statistical power and underestimate true clinical regression, whereas the combined histologic and cytologic/HPV criteria used in the main model [30] better capture real-world clinical dynamics.

#### 4.2.2. Findings from the Immediate Surgery Group

Although statistically insignificant, pathological regression was less frequently observed in patients in their 30s and those over 50 years old (Table 2, Figure 2A). Notably, among patients who underwent early intervention, the presence of HPV 16 infection and being in their 30s increased the risk of hidden progressive lesions (Table 4). Considering these findings, expectant management could be recommended for individuals aged <30 years old. This aligns with the study by Tainio et al. [15], although another study reports that expectant management is also possible for individuals in their 30s [18]. Despite the differences in age groups, these results collectively support the ASCCP guidelines, which recommend expectant management for individuals aged <25 years [27].

A prior study also reported that HPV 16 or 18 infection was associated with a higher risk of unexpected high-grade lesions on conization despite low-grade cytology [26], consistent with our results. Therefore, for women in their 30s or those with HPV 16 infection whose lesions persist beyond the median regression period, early treatment may be preferable to continued observation.

### 4.3. Strengths and Weaknesses

The strength of this study was able to recommend specific guidelines for expectant management by comparing the prognostic factors between the observation group and the early intervention group. In particular, meaningful results were obtained regarding HPV genotype-based risk stratification, which may help guide individualized surveillance strategies in clinical practice.

However, this study had several limitations. First, the inclusion of CIN 2 patients in expectant management was relatively small compared with previous studies. This is because cervical cancer had the highest incidence among gynecologic cancers in Korea up until 2019 [2], leading clinicians to choose the most aggressive treatment options within the guidelines. As a result, it is very rare in Korea to follow up on CIN 2 cases without immediate treatment. In this context, the result of 92 CIN 2 patients was considered meaningful in Korea.

Second, because this was a multicenter retrospective study, inter-laboratory variability in histopathologic interpretation and HPV testing, as well as the absence of provider-level data such as colposcopist certification, may have introduced potential selection bias and affected the precision of the findings. Nevertheless, all participating hospitals were secondary or tertiary care centers within the Catholic Medical Center network, where pathology departments are accredited by the Korean Society of Pathologists quality control program and maintain collaborative diagnostic review systems. Moreover, all procedures were performed by board-certified obstetrician-gynecologists. Therefore, a consistently high diagnostic accuracy and standard of care can reasonably be assumed.

Third, considering the ongoing debate regarding the interpretation of multiple testing and its potential impact on result credibility, we carefully considered the need for multiple testing correction following current methodological recommendations to ensure transparent and unbiased interpretation of our findings [30]. However, given the large number of HPV genotypes analyzed and the limited events for each subtype, applying a formal correction (e.g., Bonferroni or FDR) could have masked clinically meaningful associations. As the secondary objective of this study was to examine genotype-specific risks and regression tendencies of HPV infection, identifying patterns across different HPV types was considered clinically important, and results were interpreted with this focus in mind.

Fourth, previous studies have reported on the correlation between HPV infection, cervical cancer, and pelvic inflammation, particularly in the case of *Chlamydia* spp. infection [31,32].

### 4.4. Implications for Practice and Future Research

The most critical considerations in the management of CIN are the duration of lesion persistence and the identification of risk factors for progression. This study provides valuable insights into both the timing and likelihood of regression, as well as the prognostic indicators associated with unfavorable outcomes.

Although most regressions occurred within 1.5 years, annual co-testing for at least two years post-regression is advisable to avoid misclassification due to transient viral suppression or test variability. In patients with high-grade baseline cytology or those with elevated risk factors such as HPV 58 infection, extending co-testing to three years may be warranted to ensure adequate surveillance. Moreover, our observation that hematological disorders may negatively affect regression represents a relatively unexplored but potentially important area of research.

In addition, among patients with CIN, particular attention should be paid during follow-up to those infected with HPV 16 or to individuals aged 30 years or older, as these factors were associated with a higher likelihood of pathologic upgrading at surgery. Such findings suggest that while expectant management is generally safe, tailored follow-up strategies may be necessary for patients with high-risk features.

Furthermore, HPV genotypes 16 and 58 appear to represent particularly concerning subtypes in the Korean population. These results emphasize the need for genotype-specific surveillance and should be taken into consideration when formulating future prophylactic vaccination and prevention strategies at the national level. Future investigations are warranted to elucidate the biological mechanisms by which these comorbidities influence HPV persistence and CIN progression.

## 5. Conclusions

Cases classified as CIN 1 and CIN 2 showed a high likelihood of regression, supporting the feasibility of expectant management with a median duration of up to 1.5 and 1.2 years, respectively. However, the presence of high-risk HPV infection—particularly HPV 58—high-grade initial cytology, or hematological disorders (with lower ANC) may decrease the likelihood of regression. Patients with HPV 16 infection or those in their 30s exhibited an increased risk of pathological upgrading, warranting closer monitoring.

## Figures and Tables

**Table 1:** Patients’ baseline characteristics stratified by disease state.

Characteristic	N	Overall N = 561 ^a^	Persistent n = 151 ^a^	Progression n = 53 ^a^	Regression n = 357 ^a^	*p*-Value ^b^
Diagnosed age, years	561					0.3
20s		138 (25%)	36 (24%)	15 (28%)	87 (24%)	
30s		149 (27%)	41 (27%)	17 (32%)	91 (25%)	
40s		127 (23%)	27 (18%)	8 (15%)	92 (26%)	
over 50s		147 (26%)	47 (31%)	13 (25%)	87 (24%)	
Time to clinical outcome ^c^	561	568 (18, 3382)	661 (367, 3382)	656 (91, 1575)	520 (18, 1987)	<0.001
HPV virus type	531					<0.001
Low-risk		89 (17%)	32 (22%)	7 (14%)	50 (15%)	
High-risk		324 (61%)	85 (59%)	43 (84%)	196 (58%)	
Negative		118 (22%)	26 (18%)	1 (2.0%)	91 (27%)	
High-risk HPV virus						
16	531	45 (8.5%)	10 (7.0%)	10 (20%)	25 (7.4%)	0.021
18	531	13 (2.4%)	2 (1.4%)	2 (3.9%)	9 (2.7%)	0.5
52	531	27 (5.1%)	6 (4.2%)	2 (3.9%)	19 (5.6%)	0.9
58	531	38 (7.2%)	10 (7.0%)	9 (18%)	19 (5.6%)	0.015
31	531	8 (1.5%)	4 (2.8%)	2 (3.9%)	2 (0.6%)	0.029
33	531	6 (1.1%)	2 (1.4%)	2 (3.9%)	2 (0.6%)	0.083
45	531	7 (1.3%)	2 (1.4%)	1 (2.0%)	4 (1.2%)	0.7
35	531	10 (1.9%)	2 (1.4%)	2 (3.9%)	6 (1.8%)	0.4
39	531	16 (3.0%)	3 (2.1%)	1 (2.0%)	12 (3.6%)	0.8
51	531	31 (5.8%)	9 (6.3%)	3 (5.9%)	19 (5.6%)	>0.9
56	531	27 (5.1%)	12 (8.4%)	3 (5.9%)	12 (3.6%)	0.068
59	531	7 (1.3%)	3 (2.1%)	0 (0%)	4 (1.2%)	0.5
66	531	23 (4.3%)	7 (4.9%)	3 (5.9%)	13 (3.9%)	0.6
68	531	32 (6.0%)	5 (3.5%)	3 (5.9%)	24 (7.1%)	0.3
Multiple HPV infection	531	73 (14%)	23 (16%)	13 (25%)	37 (11%)	0.012
Initial Cytology	561					<0.001
ASCUS/LSIL		268 (48%)	102 (68%)	24 (45%)	142 (40%)	
ASC-H/HSIL		37 (6.6%)	8 (5.3%)	13 (25%)	16 (4.5%)	
Normal/RCC		256 (46%)	41 (27%)	16 (30%)	199 (56%)	
Hematological Disorder	561	14 (2.5%)	5 (3.3%)	4 (7.5%)	5 (1.4%)	0.022
Pelvic inflammatory disease	561	260 (46%)	73 (48%)	26 (49%)	161 (45%)	0.7

CIN, cervical intraepithelial neoplasia; HPV, human papillomavirus; RCC, reactive cellular change; ASCUS, atypical squamous cells of undetermined significance; LSIL, low-grade squamous intraepithelial lesion; ASC-H, atypical squamous cells—cannot exclude HSIL; HSIL, high-grade squamous intraepithelial lesion. ^a^ n (%); Median (Range). ^b^ Pearson’s Chi-squared test; Kruskal–Wallis rank sum test; Fisher’s exact test. ^c^ Median follow-up (days).

**Table 2:** Results of univariate and multivariate regression analyses (Clinical and Cytologic Factors).

	Univariate				Multivariate			
Characteristic	N	Crude HR	95% CI	*p*-Value	N	Adjusted HR	95% CI	*p*-Value
Initial CIN state	561				531			
CIN 1		1.00	Ref.			1.00	Ref.	
CIN 2		1.16	0.86, 1.57	0.3		1.47	1.07, 2.01	0. 016
Diagnosed age	561				531			
20s		1.00	Ref.			1.00	Ref.	
30s		0.83	0.62, 1.11	0.2		0.87	0.63, 1.21	0.4
40s		1.09	0.82, 1.45	0.6		1.03	0.76, 1.40	0.9
over 50s		0.87	0.64, 1.17	0.4		0.92	0.67, 1.27	0.6
Initial Cytology	561				531			
Normal/RCC		1.00	Ref.			1.00	Ref.	
ASCUS/LSIL		0.54	0.44, 0.67	<0.001		0.54	0.44, 0.68	<0.001
ASC-H/HSIL		0.30	0.17, 0.52	<0.001		0.30	0.17, 0.55	<0.001
Hematological Disorder	561				531			
No		1.00	Ref.			1.00	Ref.	
Yes		0.39	0.16, 0.94	0.035		0.39	0.16, 0.98	0.045
Pelvic inflammatory disease	561				531			
No		1.00	Ref.			1.00	Ref.	
Yes		0.91	0.74, 1.12	0.4		0.97	0.79, 1.21	0.8

HR, Hazard Ratio; CI, Confidence Interval; CIN, Cervical Intraepithelial Neoplasia; RCC, Reactive Cellular Change; ASCUS, Atypical Squamous Cells of Undetermined Significance; LSIL, Low-Grade Squamous Intraepithelial Lesion; ASC-H, Atypical Squamous Cells—Cannot Exclude HSIL; HSIL, High-Grade Squamous Intraepithelial Lesion.

**Table 3:** Results of univariate and multivariate regression analyses (HPV genotypes).

	Univariate				Multivariate			
Characteristic	N	Crude HR ^a^	95% CI ^a^	*p*-Value	N	Adjusted HR ^a^	95% CI ^a^	*p*-Value
HPV virus type	531				531			
Low risk or Negative		1.00	Ref.			1.00	Ref.	
High risk		0.78	0.63, 0.96	0.020		0.78	0.63, 0.97	0.025
HPV type ^a^								
16	531	0.86	0.55, 1.34	0.5	531	0.89	0.56, 1.39	0.6
18	531	0.82	0.44, 1.52	0.5	531	0.73	0.38, 1.43	0.4
52	531	1.42	0.86, 2.35	0.2	531	1.44	0.89, 2.32	0.14
58	531	0.65	0.41, 1.04	0.071	531	0.61	0.39, 0.96	0.032
31	531	0.43	0.11, 1.66	0.2	531			NA ^b^
33	531	0.41	0.11, 1.56	0.2	531	0.47	0.13, 1.76	0.3
45	531	0.75	0.29, 1.96	0.6	531	0.98	0.39, 2.49	>0.9
35	531	0.61	0.28, 1.32	0.2	531	0.71	0.34, 1.49	0.4
39	531	1.38	0.70, 2.74	0.4	531	- ^c^	- ^c^	0.2 ^c^
51	531	0.99	0.59, 1.65	>0.9	531	0.89	0.52, 1.50	0.7
56	531	0.69	0.39, 1.22	0.2	531	0.72	0.40, 1.31	0.3
59	531	0.65	0.35, 1.24	0.2	531	- ^c^	- ^c^	0.4 ^c^
66	531	0.65	0.39, 1.08	0.10	531	0.67	0.40, 1.13	0.14
68	531	1.23	0.84, 1.79	0.3	531	1.02	0.72, 1.46	0.9
HPV multiple infection	531				531			
No		1.00	Ref.			1.00	Ref.	
Yes		0.65	0.46, 0.91	0.011		0.73	0.52, 1.02	0.066

HR, Hazard Ratio; CI, Confidence Interval; HPV, Human Papillomavirus. ^a^ Multivariate analysis was performed for each HPV type. ^b^ Not available; no observations for cases with the event. ^c ^ Adjusted HR not available due to insufficient sample size. *p*-value calculated using Fisher's exact test.

**Table 4:** Factors indicative of benefits from early interventions.

Characteristic	Overall N = 359	Expected n = 236	Pathologic Upgrading at Surgery n = 123		*p*-Value ^a^
			Hidden CIN 2	Hidden CIN 3	
			n = 31	n = 92	
Preoperative biopsy result					<0.001
CIN 1	108 (30%)	61 (26%)	25 (81%)	22 (24%)	
CIN 2	236 (66%)	167 (71%)	0 (0%)	69 (75%)	
HSIL	6 (1.7%)	6 (2.5%)	0 (0%)	0 (0%)	
Negative	1 (0.3%)	1 (0.4%)	0 (0%)	0 (0%)	
Not detected	8 (2.2%)	1 (0.4%)	6 (19%)	1 (1.0%)	
Diagnosed age					0.016
20s	57 (16%)	41 (17%)	2 (6.5%)	14 (15%)	
30s	90 (25%)	50 (21%)	5 (16%)	35 (38%)	
40s	92 (26%)	65 (28%)	8 (26%)	19 (21%)	
over 50s	120 (33%)	80 (34%)	16 (52%)	24 (26%)	
HPV virus type					0.4
Negative	32 (12%)	23 (10%)	2 (6.5%)	7 (7.6%)	
Low risk	33 (12%)	17 (7.2%)	2 (6.5%)	14 (15%)	
High risk	212 (77%)	137 (58%)	20 (65%)	55 (60%)	
HPV type					
16	39 (14%)	16 (6.8%)	5 (16%)	18 (20%)	0.005
18	8 (2.9%)	6 (2.5%)	1 (3.2%)	1 (1.0%)	0.5
52	28 (10%)	16 (6.8%)	4 (13%)	8 (8.7%)	0.4
58	17 (6.1%)	11 (4.7%)	0 (0%)	6 (6.5%)	0.5
31	9 (3.2%)	5 (2.1%)	0 (0%)	4 (4.3%)	0.5
33	2 (0.7%)	2 (0.8%)	0 (0%)	0 (0%)	>0.9
35	6 (2.2%)	6 (2.5%)	0 (0%)	0 (0%)	0.3
39	4 (1.4%)	4 (1.7%)	0 (0%)	0 (0%)	0.5
51	11 (4.0%)	8 (3.4%)	2 (6.5%)	1 (1.0%)	0.2
56	3 (1.1%)	1 (0.4%)	0 (0%)	2 (2.2%)	0.4
66	2 (0.7%)	2 (0.8%)	0 (0%)	0 (0%)	>0.9
68	3 (1.1%)	3 (1.3%)	0 (0%)	0 (0%)	0.7
HPV multiple infection	41 (15%)	28 (12%)	4 (13%)	9 (9.8%)	0.7
Pelvic inflammatory disease	248 (69%)	167 (71%)	24 (77%)	57 (62%)	0.2
Hematological disorders	8 (2.2%)	6 (2.5%)	0 (0%)	2 (2.2%)	>0.9

CIN, Cervical Intraepithelial Neoplasia; HSIL, High-Grade Squamous Intraepithelial Lesion; HPV, Human Papillomavirus. ^a^ Pearson’s Chi-squared test; Fisher’s exact test.

## Data Availability

Individual participant data that underlie the results reported in this article, after de-identification, are available upon reasonable request from the corresponding author.

## Supplementary Materials

The following supporting information can be downloaded at: https://www.mdpi.com/article/10.3390/cancers17233738/s1. Table S1: The disease codes used in this study based on the International Classification of Diseases (10th revision); Figure S1: Diagnosis and prognosis flowchart of CIN 1/CIN 2 patients; Table S2: The characteristics of Hematological disorder group; Table S3: Results of multivariate regression analyses (only biopsy-confirmed cases in the expectant management group); Table S4: Results of multivariate regression analyses (≥6-month follow-up cohort).

## Abbreviations

The following abbreviations are used in this manuscript:

ANC	Absolute neutrophil count
ASC-H	Atypical squamous cells-cannot exclude high-grade squamous intraepithelial lesion
ASC-US	Atypical squamous cells of undetermined significance
CIN	Cervical intraepithelial neoplasia
CI	Confidence interval
DNA	Deoxyribonucleic acid
HPV	Human papillomavirus
HR	Hazard ratio
HSIL	High-grade squamous intraepithelial lesion
IRB	Institutional Review Board
LSIL	Low-grade squamous intraepithelial lesion
p16	Cyclin-dependent kinase inhibitor 2A
ASCCP	American Society for Colposcopy and Cervical Pathology
