# Supplementary material for: Evaluation of Risk Factors Associated with Expectant Management in CIN 1/2: A Multicenter Real-World Cohort Study
Source: Cancers (Basel). 2025 Nov 22;17(23):3738. doi: 10.3390/cancers17233738 (PMC12691198; doi:10.3390/cancers17233738)
Supplement: Supplementary file 1 [file cancers-17-03738-s001.zip › cancers-3946440-supplementary.pdf]

Table S1. The disease codes used in this study based on the International Classification of Diseases (10th revision)

Diagnosis	Disease codes
**Cervical cancer**	C530, C531 or C539.x
**Pelvic inflammatory disease**	N701, N709.x, N72.x, N730.x, N731.x, N738, N739.x, N760.x, N761.x, N762.x, N763, N764, N766.x, N768.x, and N771
**Hematological disorders - Anemia, Thrombocytopenia**	D509.x, D590, D592, D595.x, D610.x, D619.x, D62.x, D693.x, D6938.x, and D696.x
**Hematological disorders - Tumors of the hematopoietic and lymphoid tissues**	C8338.x, C8449.x, C852, C8599.x, C863, C910.x, C9108.x, C920.x, C9208.x, C921.x, C924.x, and C950.x

Figure S1. Diagnosis and prognosis flowchart of CIN 1/CIN 2 patients

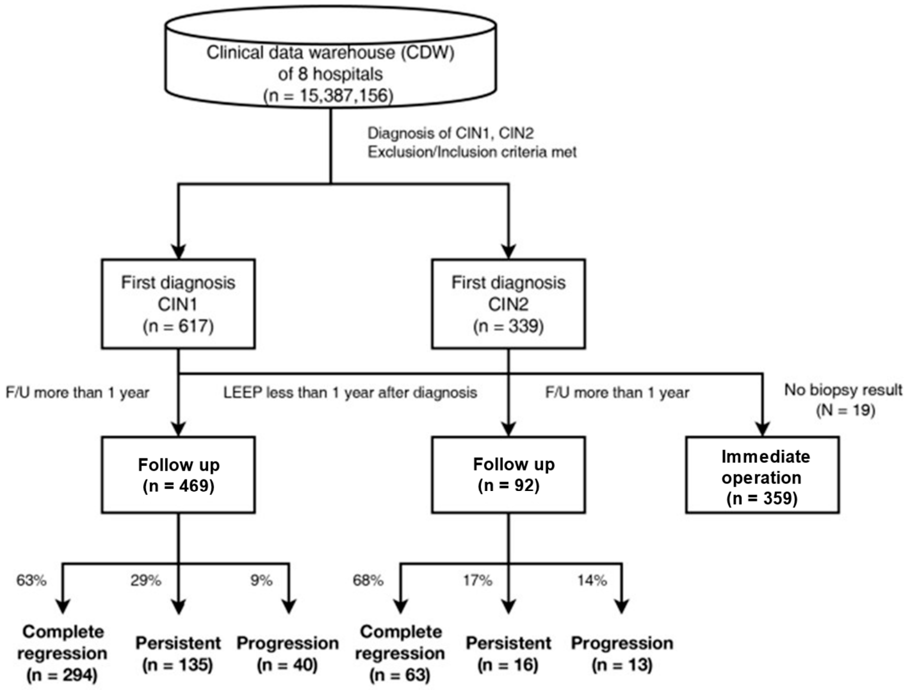

Table S2. The characteristics of Hematological disorder group

Characteristic		Overall N = 22 [a]	Bad Prognosis [b] n = 11 [a]	Good Prognosis [c] n = 11 [a]	*p*-value [d]
**Hematological disorder**	22				0.3
Nutritional anemia (D50-53)		17 (77%)	7 (64%)	10(91%)	
Hemolytic or Aplastic or Other disease (D55-89)		5(23%)	4 (36%)	1 (9.1%)	
**Hemoglobin [g/dl]**	22	12 (2)	12 (1)	11 (2)	0.04
**White blood cell (WBC) counts**	22	7,117 (3,503)	5,921 (2,996)	8,314 (3,718)	0.13
ANC (absolute neutrophil count)	20	4,386 (3,349)	3,001(1,863)	5,772 (3,992)	**0.043**
Lymphocyte [%]	20	34 (17)	41 (19)	28 (13)	0.14
**Platelet counts [x10^3]**	21	222 (99)	199 (95)	248 (102)	0.3

[a]n (%); Mean (SD)
[b]Persistent or progressive disease in the observation group / worse-than-expected outcomes in the early intervention group
[c]Regression in the observation group / expected outcomes in the early intervention group
[d]Fisher's exact test; Wilcoxon rank sum test; Wilcoxon rank sum exact test

Table S3. Results of multivariate regression analyses (only biopsy-confirmed cases in the expectant management group)

Characteristic	N	HR[1]	95% CI[1]	*p*-value
**initial CIN state**	531			
CIN1		1.00	—	
CIN2		2.93	2.06, 4.17	**<0.001**
**Diagnosed age**	531			
20s		1.00	—	
30s		0.74	0.49, 1.13	0.2
40s		0.72	0.47, 1.10	0.13
over 50s		0.59	0.36, 0.96	**0.034**
**Pelvic inflammatory disease**	531			
No		1.00	—	
Yes		1.33	0.97, 1.82	0.080
**Hematological disorder**	531			
No		1.00	—	
Yes		0.99	0.41, 2.36	>0.9
**HPV virus type**	531			
Low risk or Negative		1.00	—	
High risk		1.09	0.77, 1.53	0.6
**HPV high-risk multiple infection**	531			
No		1.00	—	
Yes		0.70	0.45, 1.11	0.13
**HPV high-risk type: 16**	531			
No		1.00	—	
Yes		0.90	0.51, 1.60	0.7
**HPV high-risk type: 18**	531			
No		1.00	—	
Yes		1.61	0.96, 2.71	0.071
**HPV high-risk type: 31**	531			*NA*[2]
**HPV high-risk type: 52**	531			
No		1.00	—	
Yes		1.53	0.81, 2.87	0.2
**HPV high-risk type: 58**	531			
No		1.00	—	
Yes		1.12	0.63, 1.96	0.7

Characteristic	N	HR[1]	95% CI[1]	*p*-value
**HPV high-risk type: 39**	531	-		0.081[3]
**HPV high-risk type: 33**	531			
No		1.00	—	
Yes		2.19	0.92, 5.24	0.077
**HPV high-risk type: 45**	531			
No		1.00	—	
Yes		1.13	0.28, 4.49	0.9
**HPV high-risk type: 35**	531			
No		1.00	—	
Yes		0.59	0.21, 1.65	0.3
**HPV high-risk type: 51**	531			
No		1.00	—	
Yes		0.77	0.39, 1.51	0.5
**HPV high-risk type: 56**	531			
No		1.00	—	
Yes		1.10	0.54, 2.27	0.8
**HPV high-risk type: 59**		-		0.5[3]
**HPV high-risk type: 66**	531			
No		1.00	—	
Yes		1.01	0.51, 2.02	>0.9
**HPV high-risk type: 68**	531			
No		1.00	—	
Yes		1.42	0.79, 2.55	0.3

[1]HR = Hazard Ratio, CI = Confidence Interval

[2]No observation when event occurred

[3]Gray's test

Table S4. Results of multivariate regression analyses (≥6-month follow-up cohort)

Characteristic	N	HR[1]	95% CI[1]	*p*-value
**initial CIN state**	588			
CIN1		1.00	—	
CIN2		1.27	0.92, 1.75	0.14
**Diagnosed age**	588			
20s		1.00	—	
30s		0.85	0.61, 1.18	0.3
40s		1.13	0.83, 1.54	0.4
over 50s		1.04	0.75, 1.44	0.8
**Pelvic inflammatory disease**	588			
No		1.00	—	
Yes		0.91	0.73, 1.14	0.4
**Hematological disorders**	588			
No		1.00	—	
Yes		0.44	0.16, 1.20	0.11
**HPV virus type**	588			
Low risk or Negative		1.00	—	
High risk		0.75	0.60, 0.93	**0.010**
**HPV high-risk multiple infection**	588			
No		1.00	—	
Yes		0.63	0.44, 0.90	**0.011**
**HPV high-risk type: 16**	588			
No		1.00	—	
Yes		0.74	0.46, 1.21	0.2
**HPV high-risk type: 18**	588			
No		1.00	—	
Yes		0.94	0.57, 1.54	0.8
**HPV high-risk type: 31**	588			*NA*[2]
**HPV high-risk type: 52**	588			
No		1.00	—	
Yes		1.44	0.86, 2.40	0.2

Characteristic	N	HR[1]	95% CI[1]	*p*-value
**HPV high-risk type: 58**	588			
No		1.00	—	
Yes		0.68	0.43, 1.09	0.11
**HPV high-risk type: 39**	588	-		0.3[3]
**HPV high-risk type: 33**	588			
No		1.00	—	
Yes		0.51	0.08, 3.09	0.5
**HPV high-risk type: 45**	588			
No		1.00	—	
Yes		0.79	0.29, 2.14	0.6
**HPV high-risk type: 35**	588			
No		1.00	—	
Yes		0.55	0.26, 1.18	0.12
**HPV high-risk type: 51**	588			
No		1.00	—	
Yes		0.83	0.48, 1.45	0.5
**HPV high-risk type: 56**	588			
No		1.00	—	
Yes		0.67	0.37, 1.22	0.2
**HPV high-risk type: 59**		-		0.3[3]
**HPV high-risk type: 66**	588			
No		1.00	—	
Yes		0.67	0.39, 1.15	0.14
**HPV high-risk type: 68**	588			
No		1.00	—	
Yes		1.18	0.79, 1.76	0.4

[1]HR = Hazard Ratio, CI = Confidence Interval

[2]No observation when event occurred

[3]Gray's test
